# Aminoacyl-tRNA-Charged Eukaryotic Elongation Factor 1A Is the *Bona Fide* Substrate for *Legionella pneumophila* Effector Glucosyltransferases

**DOI:** 10.1371/journal.pone.0029525

**Published:** 2011-12-22

**Authors:** Tina Tzivelekidis, Thomas Jank, Corinna Pohl, Andreas Schlosser, Sabine Rospert, Charlotte R. Knudsen, Marina V. Rodnina, Yury Belyi, Klaus Aktories

**Affiliations:** 1 Institut für Experimentelle und Klinische Pharmakologie und Toxikologie, Albert-Ludwigs-Universität, Freiburg, Germany; 2 Fakultät für Biologie, Albert-Ludwigs-Universität Freiburg, Freiburg, Germany; 3 Max-Planck-Institut für biophysikalische Chemie, Göttingen, Germany; 4 Zentrum für Biosystemanalyse, Core Facility Proteomics, Albert-Ludwigs-Universität Freiburg, Freiburg, Germany; 5 Institut für Biochemie und Mikrobiologie, ZBMZ, Albert-Ludwigs-Universität, Freiburg, Germany; 6 Department of Molecular Biology, University of Aarhus, Aarhus, Denmark; 7 Gamaleya Research Institute, Moscow, Russia; University of Louisville, United States of America

## Abstract

*Legionella pneumophila,* which is the causative organism of Legionnaireś disease, translocates numerous effector proteins into the host cell cytosol by a type IV secretion system during infection. Among the most potent effector proteins of *Legionella* are glucosyltransferases (lgt's), which selectively modify eukaryotic elongation factor (eEF) 1A at Ser-53 in the GTP binding domain. Glucosylation results in inhibition of protein synthesis. Here we show that *in vitro* glucosylation of yeast and mouse eEF1A by Lgt3 in the presence of the factors Phe-tRNA^Phe^ and GTP was enhanced 150 and 590-fold, respectively. The glucosylation of eEF1A catalyzed by Lgt1 and 2 was increased about 70-fold. By comparison of uncharged tRNA with two distinct aminoacyl-tRNAs (His-tRNA^His^ and Phe-tRNA^Phe^) we could show that aminoacylation is crucial for Lgt-catalyzed glucosylation. Aminoacyl-tRNA had no effect on the enzymatic properties of lgt's and did not enhance the glucosylation rate of eEF1A truncation mutants, consisting of the GTPase domain only or of a 5 kDa peptide covering Ser-53 of eEF1A. Furthermore, binding of aminoacyl-tRNA to eEF1A was not altered by glucosylation. Taken together, our data suggest that the ternary complex, consisting of eEF1A, aminoacyl-tRNA and GTP, is the *bona fide* substrate for lgt's.

## Introduction

*Legionella pneumophila* is responsible for severe pneumonia referred to as Legionnaireś disease [1]. The bacterium lives ubiquitously in aquatic environments, where it invades and replicates within protozoa [2]. Infection occurs predominantly due to inhalation of contaminated water sources [3]. After entering phagocytic host cells such as alveolar macrophages, *Legionella* survives and replicates intracellularly [4]. To this end, *Legionella* produces a plethora of effector proteins, which are injected into the host cell cytosol via a type IVb secretion system (Dot/Icm system) [5]–[7] to subvert the phagosome into a specialized compartment known as “*Legionella* containing vacuole” (LCV) [8]–[10]. The LCV fails to enter the lysosomal degradative pathway and rather generates an intracellular environment where *Legionella* can replicate extensively. Bioinformatics, genetic and reporter-fusion screens for Dot/Icm effector proteins of *Legionella* identified >300 candidates [8], [11]–[15]. Only a few were biochemically validated and even less were characterized at the molecular level [8]. Some of these effectors are known to target distinct regulatory host cell factors such as GTPases or ATPases [16]–[23] in order to transform the host cell into a replication permissive environment [8]. A number of effectors interact with vesicles and influence their maturation by altering phosphoinositides involved in trafficking and regulation [24]. However, most effector targets remain elusive.

Among the best-studied effectors are members of the *Legionella* glucosyltransferase family (Lgt) [25], [26]. Pathogenic strains of *L. pneumophila* possess up to three Lgt isoforms (Lgt1- 3) [27]. Unfortunately, data describing *lgt*-deletion strains concerning intracellular replication or phenotypic alteration are lacking. Recently, the crystal structure of Lgt1 was solved independently by two groups [28], [29]. Lgt1 exhibits a typical glucosyltransferase GT-A type of fold with a central UDP-glucose binding domain, sharing significant similarity with the glucosyltransferase domain of *Clostridium difficile* toxins A and B [26], [30]. Inside the host cell, lgt's modify eukaryotic elongation factor 1A (eEF1A) by mono-*O*-glucosylation and thereby inhibit protein synthesis. It has been suggested that inhibition of protein synthesis of host cells by lgt's induces cell death. This might be important at the end of the proliferation cycle of *Legionella* supporting the release of the pathogen [31]. However, it cannot be excluded that inhibition of protein synthesis is necessary for establishment of the optimal environment for *Legionella* replication. Moreover, protein synthesis inhibition by *L. pneumophila* may have major consequences for the innate immune response of the host [32].

Eukaryotic EF1A, one of the most abundant proteins in the cytosol of the eukaryotic cell, is glucosylated at Ser-53, which is located on a protruding region including helices A* and Á' in the GTPase domain [31]. This region is lacking in the prokaryotic homolog of eEF1A, EF-Tu and therefore, *Legionella* is protected from its own toxic effector. The canonical function of eEF1A is the delivery of aminoacyl-tRNAs to the ribosome for protein synthesis. eEF1A belongs to the superfamily of the GTP binding proteins, which can bind and hydrolyze GTP. Upon GTP-hydrolysis, the conformation of the eEF1A switches between an active (GTP-bound) and inactive (GDP-bound) state. The eEF1A cycle begins with the GDP/GTP exchange catalyzed by the guanine nucleotide exchange factor eEF1Bα/β. In the GTP-bound form eEF1A has high affinity for aa-tRNA and forms a stable ternary complex [33]. The ternary complex protects aa-tRNA from RNases and spontaneous hydrolysis and facilitates delivery of aa-tRNA to the decoding site of the ribosome (A site) [33]. eEF1A mediates the accurate interaction of aa-tRNA anticodon with the codon of the mRNA in the A site of the ribosome. Codon-anticodon recognition triggers GTP-hydrolysis by eEF1A, which might affect the affinity for eEF1A to aminoacyl-tRNA. Consequently eEF1A dissociates and enters into a new elongation cycle. Apart from the role in protein synthesis, several non-canonical functions of eEF1A have been described (e.g. nuclear export activities, turnover of misfolded proteins, actin cytoskeleton organization and cellular stress responses [34], [35].

Recently, it was shown that lgt's are able to glucosylate a decapeptide (_50_GKG**S**FKYAWV_59_) covering the loop formed by helices A* and Á' of eEF1A [36]. Interestingly, glucosylation of this decapeptide was far more efficient than the modification of purified full length eEF1A, suggesting that a specific conformation of eEF1A is the preferred substrate of Lgt. Here we show that the ternary complex conformation of eEF1A consisting of eEF1A, aminoacyl-tRNA, and GTP is the competent substrate of lgt's.

## Results

### eEF1A is glucosylated during Legionella infection of macrophages

**Figure 1:**
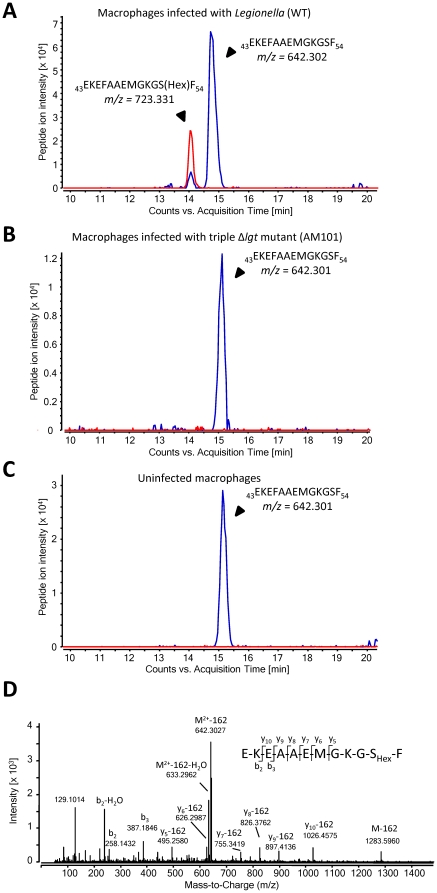
eEF1A is glucosylated during *L. pneumophila* infection at Ser53. Mammalian eEF1A was isolated by eEF1Bα-affinity chromatography from RAW 264.7 macrophages infected with *L. pneumophila* wild type (A), *L. pneumophila* triple Δ*lgt*-mutant AM101 (B) and uninfected cells (C). LC-MS/MS (Q-TOF) analysis revealed the extracted ion chromatograms shown. The peak at m/z = 642.3 (2+) belongs to the chymotryptic peptide 43-EKEFAAEMGKGSF-54 of eEF1A without modification. This was identified by MS/MS with a Mascot peptide Score of 72. The peptide from *Legionella* infected cells is partially shifted to m/z = 723.3 (2+) indicating modification by hexose (162.053 Da). The chromatograms were scanned for peptides with m/z = 642.302±10 ppm (2+) (shown in blue) and peptides with m/z = 723.331±10 ppm (2+) (shown in red). The hexose-modified form of the peptide EKEFAAEMGKGSF was exclusively detected in *Legionella* infected cells. Partial neutral loss of dehydrohexose (162.053 Da), typical for hexose-modified peptides, is observed already without additional collision energy in the MS scan (see Figure 1A, blue peak at 14 minutes). (D) Collision-induced dissociation MS/MS spectrum of the glucosylated peptide EKEFAAEMGKGSF (precursor m/z = 723.3 (2+); peak intensity = 2.5×10^4^; retention time 14.06 min) identified Ser-53 as the acceptor amino acid for glucosylation (Mascot peptide Score of 60).

First, we wanted to show, that eEF1A is glucosylated during *Legionella* infection. Therefore, we infected RAW 264.7 macrophages with wild type *Legionella pneumophila* and a mutant deficient in all *Legionella* glucosyltransferase genes (AM101) [37]. eEF1A was extracted and purified by the tight interaction with its guanine nucleotide exchange factor eEF1Bα purified in *E. coli* and applied to mass spectrometric analysis (Fig. 1). Previous analyses revealed that the interaction of eEF1A with eEF1Bα is not altered by glucosylation (data not shown). Infection with wild type *Legionella* (Fig. 1A), but not with the triple Δ*lgt* mutant (AM101) (Fig.1B) caused modification of eEF1A by a hexose moiety. MS/MS analysis revealed Ser-53 as acceptor amino acid and confirmed recent *in vitro* data [31]. We observed that the extent of glucosylation was rather high as compared to *in vitro* experiments with purified components, which is usually ∼1% [36]. Therefore, we hypothesized that additional cellular factors are essential to obtain efficient modification of eEF1A by lgt's.

### Identification of aa-tRNA and GTP as factors stimulating eEF1A glucosylation

**Figure 2:**
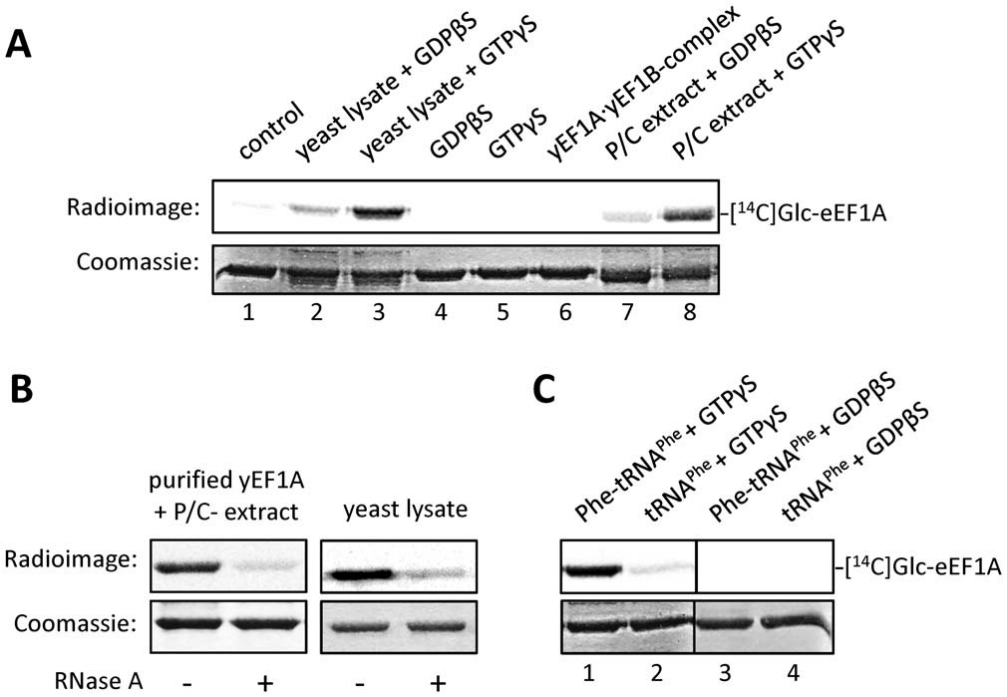
Analysis of yeast eEF1A (yEF1A) glucosylation by Lgt3. (A) Glucosyltransferase reaction was performed with Lgt3 (140 nM) and yeast eEF1A (3 µM) with UDP-[^14^C]glucose (10 µM) for 15 min at 30°C in the presence of either GDPβS (75 µM) or GTPγS (75 µM) and the following components: purified yeast eEF1A (control, lane 1), yEF1A with freshly prepared yeast cell lysate (30 µg) (lane 2 and 3), yEF1A in the presence of GDPβS (75 µM) (lane 4) or GTPγS (75 µM) (lane 5), yEF1A·yEF1Bα complex (3 µM each) (lane 6), yEF1A with 5 µl phenol/chloroform extract (P/C extract) prepared from yeast lysate (lane 7 and 8). Prior to glucosyltransferase reaction, the mixtures were preincubated for 10 min at RT. Products were separated by SDS-PAGE and glucosylated eEF1A was visualized by autoradiography. (B) Effect of the RNase A treatment. Glucosylation of 3 µM eEF1A by Lgt3 (140 nM) in yeast lysate or with P/C-extract was performed after treatment with RNase A (1.6 mg/ml) for 10 min at RT. (C) Glucosyltransferase reaction was performed as in (A) with the addition of Phe-tRNA^Phe^ or uncharged yeast tRNA^Phe^ (1 µM) in the presence of GDPβS (75 µM) or GTPγS (75 µM), respectively.

To search for additional cytosolic factors, we used Lgt3 as a model glycosyltransferase, because it exhibits the highest inhibitory potency on protein synthesis *in vitro* among the three lgt's [27]. We first tested whether the extent of glucosylation of purified yeast eEF1A was affected by the addition of freshly prepared yeast lysate (Fig. 2). Whereas modification of purified yeast eEF1A was hardly detected (Fig. 2A, lane 1), the addition of freshly prepared yeast lysate in combination with the non-hydrolysable GTP analogue GTPγS, but not with the GDP analogue GDPβS, strongly enhanced glucosylation (see Fig. 2A, lane 2 and 3). The same effect was observed with GTP and GDP (data not shown). The addition of GTPγS or GDPβS in the reaction mixture without yeast cell extract had no effect (Fig. 2A, lane 4 and 5). Therefore, we concluded that an additional factor present in cell extracts is required. eEF1Bα, the guanine nucleotide exchange factor of eEF1A was shown to form a tight complex with eEF1A [38], [39]. However, Lgt3-induced glucosylation could not be detected with the purified eEF1A•eEF1Bα complex (Fig. 2A, lane 6).

To isolate the supposed factor from yeast cell lysate that activated the glucosylation, we used cell fractionation. When we depleted yeast cytosol fractions from proteins by phenol/chloroform extraction and added the extract (P/C extract) to the glucosyltransferase reaction, the glucosylation of eEF1A was restored. The effect was slightly increased by GDPβS (Fig. 2A, lane 7) but strongly increased in the presence of GTPγS (Fig. 2A, lane 8). Preincubation of yeast P/C extract or yeast cell extract with RNase A impaired Lgt3-catalyzed glucosylation of eEF1A (Fig. 2B). All these results suggested a RNA component as the stimulating factor for the reaction. As aa-tRNA, in addition to GTP is a well-known eEF1A ligand, we tested the effect on the glucosyltransferase reaction of purified yeast tRNA^Phe^ and Phe-tRNA^Phe^ (Fig. 2C). Strikingly, eEF1A-glucosylation was strongly enhanced by the addition of Phe-tRNA^Phe^ in combination with GTPγS (Fig. 2C, lane 1). Uncharged tRNA^Phe^ could not stimulate glucosylation (Fig. 2C, lane 2) and also Phe-tRNA^Phe^ in combination with GDPβS was not able to stimulate glucosylation (Fig. 2C, lane 3).

**Figure 3:**
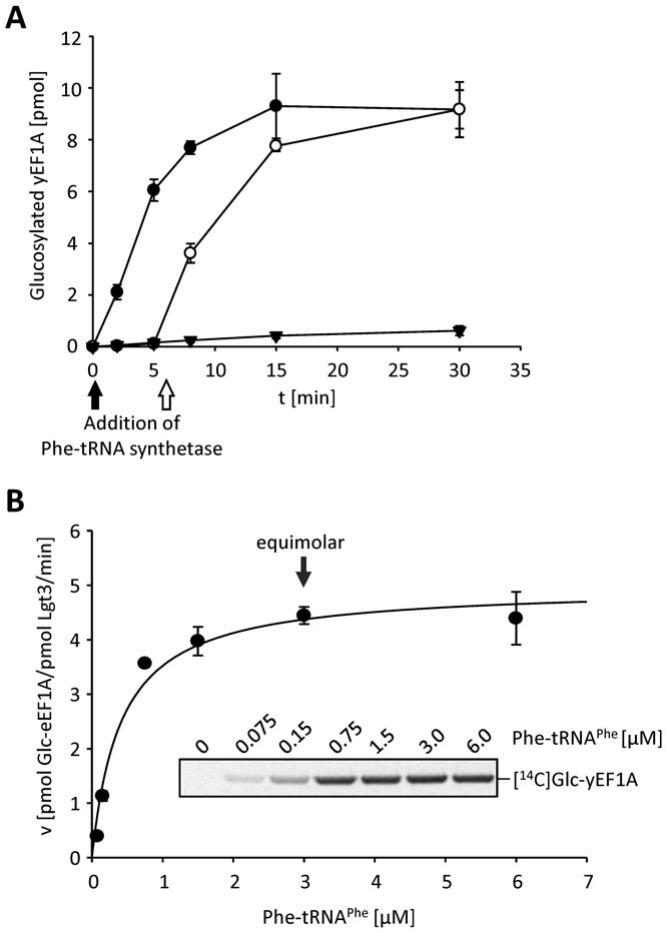
Aa-tRNA stimulates eEF1A glucosylation. (A) Time courses of the glucosylation of yeast eEF1A (yEF1A) with Lgt3 were performed in the presence of yeast tRNA (10 µM) and 10 µM UDP-[^14^C]glucose (triangles). Phe-tRNA synthetase was added at time point zero (black circles) or after 6 min (open circles) as marked by corresponding arrows. ^14^C-glucosylation of yEF1A was determined by SDS-PAGE and autoradiography. Shown curves represent means (±SD) of three independent experiments. (B) Glucosylation of yEF1A (3 µM) by Lgt3 (5 nM) was conducted with increasing concentrations of HPLC-purified [^14^C]Phe-tRNA^Phe^. Initial glucosylation rates were determined after incubation for 10 min at 30°C in the presence of 50 µM GTPγS, 10 mM PEP, 0.1 mg/ml pyruvate kinase and 10 µM UDP-[^14^C]glucose as the donor substrate. Radiolabeled aa-tRNA could be distinguished from ^14^C-glucosylated yEF1A by separation of the products by SDS-PAGE. Data shown represent velocities of yEF1A glucosylation and are given as means (±SD) of three independent experiments.

To confirm the finding that aa-tRNA but not uncharged tRNA or any additional contaminant in the reaction mixture facilitates glucosylation, we monitored the dependence of the reaction on the addition of phenylalanine tRNA-synthetase (PheRS). Efficient Lgt-induced glucosylation of eEF1A depended on the presence of PheRS, suggesting the requirement of synthesis of Phe-tRNA^Phe^ (Fig. 3A), whereas in the absence of PheRS, eEF1A glucosylation was hardly detectable. These data suggested that binding of aa-tRNA to eEF1A induces a conformational change, which allows efficient glucosylation by Lgt3.

Next, we tested whether catalytic amounts of aa-tRNA would be sufficient to induce glucosylation of eEF1A by Lgt3. The rate of glucosylation of yeast eEF1A increased with the addition of increasing amounts of HPLC-purified Phe-tRNA^Phe^ and reached maximum velocity at 3 µM, the equimolar ratio of aa-tRNA and eEF1A (Fig. 3B). These results strongly suggested that the aa-tRNA·GTP·eEF1A ternary complex, rather than eEF1A alone, is the *bona fide* substrate of the Lgt.

**Figure 4:**
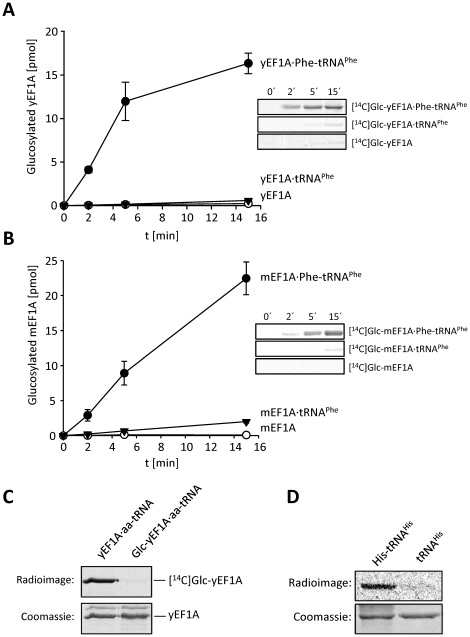
Kinetics of eEF1A glucosylation by Lgt3. Time courses of glucosylation of purified yeast eEF1A (yEF1A) (A) or mouse eEF1A (mEF1A) (B) by Lgt3 in the absence (triangles) or presence of Phe-tRNA^Phe^ (filled circles) or uncharged tRNA^Phe^ (open circles). The glucosylation reactions and analyses were performed under the conditions as described in Figure 1. Values of ^14^C-glucose incorporation are shown as the mean (±SD) of at least three independent experiments. The inserts show representative radioimages. (C) eEF1A ternary complex is quantitatively glucosylated by Lgt3. Yeast eEF1A (20 µM) was glucosylated by Lgt3 (5 µM) with Phe-tRNA^Phe^ (20 µM) and GTP (75 µM) in the absence (lane 1) or presence (lane 2) of cold UDP-glucose (1 mM). Modified and unmodified yEF1A were separated by His-eEF1Bα affinity purification as described in Materials and Methods. Thereafter, the isolated eEF1A (3 µM each) was subjected to second glucosylation reaction with radiolabeled UDP-[^14^C]glucose in presence of Phe-tRNA^Phe^ (1 µM) and GTP (75 µM). The Coomassie gel and the autoradiogram are shown. (D) Glucosylation of yEF1A (2 µM) was performed in the presence of His-tRNA^His^ or uncharged yeast tRNA with Lgt3 (140 nM).

To quantify the stimulatory effect of the ternary complex on Lgt3-catalyzed glucosylation of eEF1A, we compared initial velocities of the reaction with yeast eEF1A in the presence and absence of Phe-tRNA^Phe^ and tRNA^Phe^ (Fig. 4A). Only the eEF1A·GTP·Phe-tRNA^Phe^ ternary complex was effectively modified. The *k*_cat_-value for glucosylation of yeast ternary complex was 150-fold higher compared to yeast eEF1A incubated with Phe-tRNA^Phe^ and GDP (Table 1). Thus, the addition of aa-tRNA and GTP enabled a quantitative glucosylation of eEF1A. This was proven by glucosylation of the ternary complex of yeast eEF1A with unlabeled UDP-glucose followed by purification of the modified eEF1A by affinity chromatography and subsequent second Lgt3-induced glucosylation with radiolabeled UDP-glucose (Fig. 4C).

**Table 1:** *k*_cat_-values for glucosylation of eEF1A ternary complex by Lgt's.

Transferase	Substrate	k_cat_ [h^−1^]
Lgt3	yEF1A•GTP•Phe-tRNA^Phe^	381.7±26.7
Lgt3	yEF1A•GDP•Phe-tRNA^Phe^	2.5±0.1
Lgt3	mEF1A•GTP•Phe-tRNA^Phe^	340.5±19.5
Lgt3	mEF1A•GDP•Phe-tRNA^Phe^	0.6±0.1
Lgt1	yEF1A•GTP•Phe-tRNA^Phe^	128.2±19.9
Lgt1	yEF1A•GDP•Phe-tRNA^Phe^	1.7±0.1
Lgt2	yEF1A•GTP•Phe-tRNA^Phe^	33.5±0.9
Lgt2	yEF1A•GDP•Phe-tRNA^Phe^	0.5±0.1

Yeast eEF1A (yEF1A) and mouse eEF1A (mEF1A) were incubated for 10 min with an equimolar concentration of HPLC-purified Phe-tRNA^Phe^ and GTPγS or GDPβS (50 µM) as indicated. Afterwards the complexes were subjected to *in vitro* glucosyltransferase reactions with UDP-[^14^C]glucose (10 µM) and 5 nM Lgt1, 2 and 3. The data were autoradiographically quantified using ImageQuant. Data are given as the mean (±SD) of triplicates of 5 min time points.

To analyze the effect of Phe-tRNA^Phe^ on Lgt-catalyzed glucosylation of mammalian EF1A, we purified native eEF1A from mouse liver. The glucosylation of mouse eEF1A was even more prominent and enhanced 590-fold in the presence of aa-tRNA and GTP (Fig. 4B and Table 1).

Because the data emphasized the essential role of amino acid attached tRNA in enhancing glucosylation of eEF1A, we tested whether the effect could be reproduced in the presence of aa-tRNA other than Phe-tRNA^Phe^. We prepared His-tRNA^His^ using recombinant histidyl-tRNA-synthetase, allowed ternary complex formation with yeast eEF1A and GTPγS and analyzed the complex in glucosyltransferase reactions with Lgt3. His-tRNA^His^ stimulated glucosylation of yeast eEF1A very efficiently and the degree of stimulation was similar to that of Phe-tRNA^Phe^ (Fig. 4D).

Next, we tested the other known *Legionella* glucosyltransferases (Lgt1 and 2) with the ternary complex of yeast eEF1A and determined the initial velocities with Phe-tRNA^Phe^ in the presence of GTP or GDP. In line with Lgt3, the *k*_cat_-values for Lgt1 and 2 increased from 1.7±0.1 to 128.2±19.9 h^−1^ and 0.5±0.1 to 33.5±0.9 h^−1^, respectively, when yeast eEF1A·GTP·Phe-tRNA^Phe^ ternary complex was used as a substrate (Table 1). These results support the notion that eEF1A ternary complex is the preferred substrate for *Legionella* glucosyltransferases.

### Full length eEF1A is essential for aa-tRNA-dependent stimulation of glucosylation

**Figure 5:**
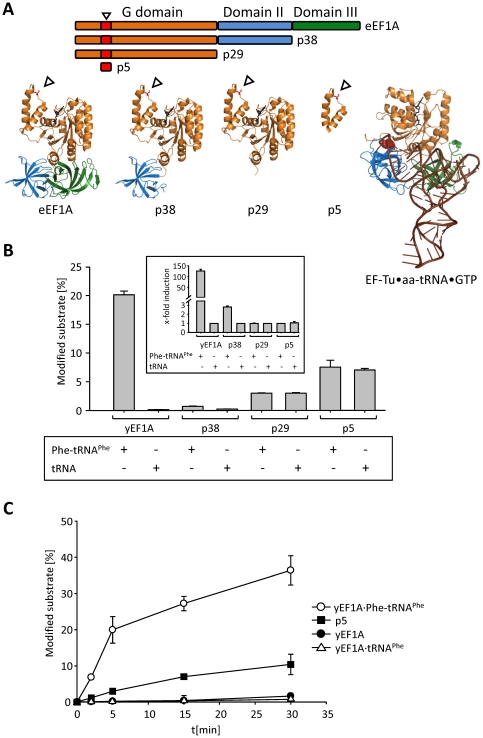
Models of eEF1A constructs and activity of Lgt's in glucosylation of eEF1A fragments. (A) Schematic representation of the eEF1A truncations used (upper panel). Lower panel: Structural models deduced from the crystal structure of eEF1A in complex with eEF1B (pdb 1IJF). For visualization of the region of aminoacyl-tRNA binding the crystal structure of the ternary complex of EF-Tu•GTP•Phe-tRNA^Phe^ (pdb 1TTT) is depicted on the right. The GTPase domain (G domain) is shaded in orange, domain II in blue, domain III in green, and tRNA is shown in brown. The aminoacyl residue of aa-tRNA located between the G domain and domain II is shown as red spheres. GDP or GTP is depicted as black sticks. The position of glucosyl acceptor serine 53 is shown in red and marked with arrowheads. The figures were prepared using PyMOL (www.pymol.org). (B) Glucosylation of different truncation fragments of yeast eEF1A (yEF1A) (3 µM) was performed with Lgt3 (140 nM) in the presence of Phe-tRNA^Phe^ or uncharged tRNA^Phe^ (each 1 µM), respectively. Constructs used: p38 represent eEF1A without domain III (aa1-349), p29 harbors the G domain (aa1-265), and p5 is a 5 kDa-peptide, comprising the helix-loop-helix region (aa29-73) of yEF1A. Glucosylation was performed under standard conditions for 15 min at 30°C. The insert shows the induction of Lgt3-catalyzed glucosylation of the fragments by Phe-tRNA^Phe^. (C) Time courses of *in vitro* glucosylation of yEF1A in complex with GTP and Phe-tRNA^Phe^ (open circles), the 45 amino acid fragment of yEF1A p5 (filled squares), yEF1A (filled circles), and yEF1A with uncharged tRNA^Phe^ (open triangles) by Lgt3. All data given represent means (±SD) of three independent experiments.

The crystal structure of the ternary complex of eEF1A with GTP and aa-tRNA is not known, but it is assumed that the interaction is similar to that found in the bacterial homolog complex [40]. The interaction surface of EF-Tu with aa-tRNA covers mainly domain II and domain III (Fig. 5A, right structure). The aminoacyl terminus of aa-tRNA is bound in between the G domain and domain II (Fig. 5A, red spheres) [41], [42]. To test whether isolated fragments of eEF1A are sufficient to bind aa-tRNA and thereby enhance its glucosylation we constructed several yeast eEF1A truncation mutants (Fig. 5A). The proteins p38 (eEF1A without domain III (aa1-349)), p29 (G domain (aa1-265)) and p5 (5 kDa peptide, comprising the helix-loop-helix region (aa29-73)) were purified and tested as substrates in the presence and absence of aa-tRNA (Fig. 5B). Except for the full-length eEF1A, only p38, which harbors the G domain and domain II, was more efficiently modified in the presence of aa-tRNA than in its absence (Fig. 5B insert), albeit to a much lower extent than the wild type eEF1A. Thus, full length eEF1A and an intact interface of the G domain and domain II seem to be necessary to allow aa-tRNA-dependent glucosylation. Belyi and co-workers demonstrated that in the absence of aa-tRNA, peptide fragments of eEF1A of a size down to 45 amino acid residues (aa29-72, fragment p5) or even a 10 amino acid fragment (aa50-59) were more efficiently glucosylated than purified full length eEF1A [36]. However, in direct comparison the ternary complex of eEF1A is more efficiently modified in the presence of aa-tRNA than the smaller fragment p5 (Fig. 5C) and is thus the preferred substrate of the reaction.

### Aminoacyl-tRNA does not directly interact with Legionella glucosyltransferases

The fact that glucosylation of the eEF1A peptide fragments p5 or p29 were not influenced by the addition of aa-tRNA ruled out the possibility that aa-tRNA stimulated the glucosyltransferase directly (Fig. 5B). In addition, we were not able to precipitate [^14^C]Phe-tRNA^Phe^ by Lgt3 (not shown). Thus, we could not detect a direct effect of aa-tRNA on the enzyme activity in the absence of eEF1A.

### The ternary complex formation is not generating additional acceptor sites for glucosylation

**Figure 6:**
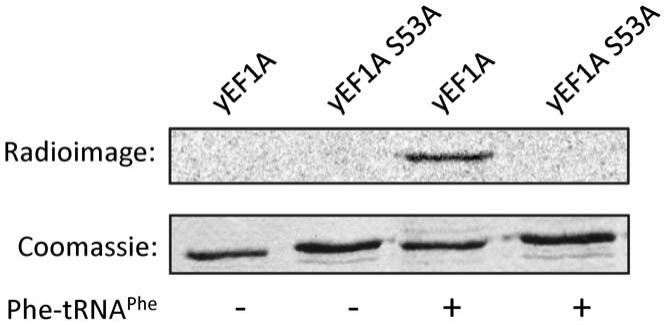
Ser-53 in eEF1A is the unique glucosylation site in the presence of Phe-tRNA^Phe^. Wild type yeast eEF1A (yEF1A) and the yeast eEF1A S53A mutant (yEF1A S53A; each 1 µM) were subjected to an *in vitro* glucosylation reaction with Lgt3 (140 nM) in the presence or absence of Phe-tRNA^Phe^.

To exclude that aa-tRNA binding generated new glucosylation sites in eEF1A in addition to Ser-53, we tested the modification with a S53A mutation of eEF1A (Fig. 6). The mutation abolished the glucosylation by Lgt3 in the presence or absence of aa-tRNA. These results indicated that aa-tRNA binding does not unmask additional glycosyl acceptor sites apart from Ser-53.

### Glucosylation does not impair the interaction between eEF1A and aa-tRNA

**Figure 7:**
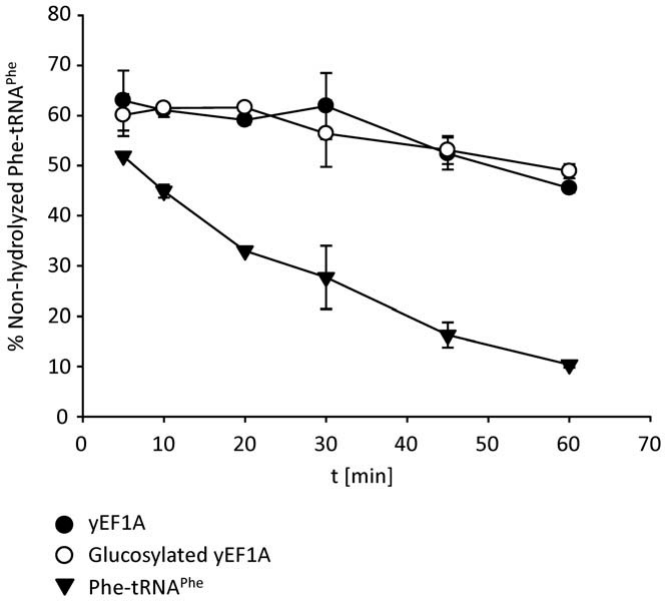
Effect of glucosylation on the stability of the ternary complex. Aminoacyl-tRNA stability towards hydrolysis was determined with 3 µM yeast eEF1A (yEF1A, filled circles) or glucosylated yeast eEF1A (yEF1A, open circles), 1 mM GTP and 1 µM [^14^C]Phe-tRNA^Phe^ for 60 min. As a control [^14^C]Phe-tRNA^Phe^ was incubated without eEF1A (triangles). Non-hydrolyzed [^14^C]Phe-tRNA^Phe^ was quantified by filter binding assay and scintillation counting.

To study whether Lgt3-catalyzed glucosylation of eEF1A has any effects on the binding of aa-tRNA to eEF1A, we performed an aa-tRNA protection assay [33]. Here, eEF1A protects aa-tRNA from spontaneous deacylation within the ternary complex. We monitored deacylation with radiolabeled [^14^C]Phe-tRNA^Phe^ in a time course at 37°C. Non-hydrolyzed [^14^C]Phe-tRNA^Phe^ was quantified after filter-binding by scintillation counting (Fig. 7). Both native and glucosylated eEF1A protected aa-tRNA from hydrolysis, whereas non-complexed aa-tRNA was hydrolyzed almost completely after 60 min of incubation. Thus, glucosylation does not inhibit the formation of the ternary complex eEF1A with GTP and aa-tRNA.

## Discussion

The members of the *Legionella* glucosyltransferase family (Lgt 1–3) modify eEF1A and potently inhibit eukaryotic protein synthesis *in vivo*. However, in contrast to the high biological activity of the enzymes, rate and efficiency of *in vitro* glucosylation of the purified eEF1A by recombinant lgt's are rather low. Notably, it was shown that a 45aa peptide, comprising aa29-72 of eEF1A, is more efficiently modified than full length eEF1A [36], suggesting that a specific conformation of eEF1A is essential for glucosylation.

Extensive search for additional factors in yeast cell lysate, which enhanced the efficiency of Lgt-catalyzed glucosylation of eEF1A, revealed non-proteinaceous cytosolic components, comprising aminoacyl-tRNA and GTP. A direct effect of aa-tRNA and GTP on the glucosyltransferase was excluded, because aa-tRNA was not able to stimulate Lgt-induced glucosylation of a 5 kDa-peptide substrate, consisting of the helix-loop-helix region of eEF1A. This indicates that the interaction of the full-length eEF1A is necessary for induction of the conformational state of eEF1A, which allows efficient glucosylation. Moreover, we observed that an equimolar ratio of aa-tRNA and eEF1A is required to obtain the maximum rate in glucosylation. This suggests that the ternary complex, consisting of eEF1A, GTP and aa-tRNA, is the competent substrate for lgt's. Kinetic measurements of glucosylation of yeast and mammalian EF1A by Lgt3 revealed that *k*_cat_-values increased 150 to 580-fold in the presence of aa-tRNA and GTP.

The interaction of the prokaryotic paralogue EF-Tu with aa-tRNA is well studied and its affinity seems to be similar for all aa-tRNAs. This general affinity was shown to depend on both sides, the nature of the esterified amino acid and the tRNA body, resulting in comparable affinity by “thermodynamic compensation” [43]–[48]. Our results highlight the importance of amino acids attached to tRNA, because uncharged tRNA was ineffective in stimulation of eEF1A glucosylation. Using histidyl-tRNA and phenylalanyl-tRNA, respectively, in complex with yeast eEF1A and GTP, we showed that the attached individual amino acid and the difference in the acceptor stem region, which is elongated by one base pair in the case of His-tRNA^His^, are not crucial for glucosylation. Of course, we cannot exclude that other types of aa-tRNA show specific effects on activation of glucosylation not observed with Phe-tRNA^Phe^ or His-tRNA^His^.

Gromadski *et al*. investigated the affinity of Phe-tRNA^Phe^ for eEF1A•GTP and obtained a *K*_d_-value of 3 nM, whereas binding of Phe-tRNA^Phe^ to the GDP-bound eEF1A was not measurable [33]. The affinity of GTP or GDP to eEF1A was comparable and their binding was suggested not to induce major structural rearrangements at least in the structural complex with eEF1Bα [39]. These observations are consistent with the glucosylation behavior of Lgt3. Neither the GTP- nor the GDP-bound form of yeast eEF1A was an efficient substrate for glucosylation, indicating failure to generate the correct target conformation. The same was true for uncharged tRNA, which reportedly has a very low affinity to GTP-bound eEF1A [33]. Thus, the mixture of eEF1A, GTP or GDP and uncharged tRNA was not efficiently modified by Lgt3.

As our results demonstrate that addition of aa-tRNA and GTP (or GTPγS) to *in vitro* enzymatic assays largely increases the rate and efficiency of glucosylation of eEF1A by lgt's, it is likely that major conformational rearrangements of eEF1A occur upon the binding of aa-tRNA and GTP, which form a stable ternary complex (eEF1A•GTP•aa-tRNA). We suggest that such an interaction induces structural changes in the G domain, particular in the helix-loop-helix region, harboring acceptor amino acid Ser-53, which allow efficient transfer of the glucose moiety to the substrate. The recently published crystal structure of archaeal EF1A from *Aeropyrum pernix* in complex with Pelota, an interaction partner recognizing stalled ribosomes containing defective mRNA, gave first insights into putative structural rearrangements of the G domain of eEF1A [49]. Pelota was shown to mimic charged aa-tRNA structurally and its binding to archaeal EF1A induces remarkable changes in the helix A*-helix Á' region, involving the conserved putative glucosyl acceptor amino acid (this is Ser-52 in the case of this organism).

Analysis of the functional protection of aa-tRNA by interaction with eEF1A revealed that glucosylation at Ser-53 of eEF1A does not influence the formation as well as the stability of the ternary complex (see Fig. 7). This suggests that the interaction of glucosylated eEF1A with aa-tRNA is well preserved under these conditions. Therefore, we speculate that inhibition of protein biosynthesis, which occurs as a result of glucosylation of eEF1A, takes place directly at the ribosome.

The functional consequences of eEF1A glucosylation and inhibition of protein synthesis during the infection process of *Legionella* is not completely understood. It might be that inhibition of protein synthesis is essential for *Legionella*-induced cell death and pathogen release after replication [29], [31]. Another possibility is the involvement of lgt's in “fine tuning” of the host metabolism to optimize the host environment for replication. Also protein synthesis inhibition might cause an altered host cell defence mechanism by reduced or elevated factors of the innate immune system [32], [50] or an altered host stress response [35], [51].Moreover, modification of the non-canonical functions of eEF1A (e.g. influence on the actin cytoskeleton or on subcellular organization [34]), which are are still controversely discussed, might also play an important role in the infection process. In conclusion, our data suggest that full length eEF1A binds aa-tRNA and GTP to form the competent substrate conformation required for glucosylation by lgt's. By selecting the ternary complex of eEF1A, aa-tRNA and GTP as the preferred substrate for Lgt-induced modification within the abundant pool of eEF1A inside cells, the bacterial effectors increase their efficiency to inhibit protein synthesis of their hosts.

## Materials and Methods

### Materials and plasmids

*Escherichia coli* strain BL21 (DE3) and *Saccharomyces cerevisiae* MH272-3fα (*ura3 leu2 his3 trp1 ade2*) were used for protein expression. *L. pneumophila* serogroup 1 Philadelphia I and the strain *L. pneumophila* AM101 deficient in all *lgt* genes (Δ*lgt*1, Δ*lgt*3, *lgt*2::*kan^r^*) were used for infection studies. The AM101 strain was kindly provided by Dr. Craig Roy (Yale University, New Haven, USA). Murine RAW 264.7 macrophages were used for infection studies. Expression vector pGEX-4T was from GE Healthcare (Freiburg, Germany), pET28a vector from Novagen (Madison, WI). pBC KS (+) and pBluescript KS (+) vectors were from Stratagene (Waldbronn, Germany). For expression of yeast proteins pRS313, pRS423, and YCpLac22 vectors were used. DNA-modifying enzymes were purchased from Fermentas (St. Leon-Rot, Germany). UDP-[^14^C]glucose was from American Radiolabeled Chemicals (St. Louis, USA). PfuII Turbo DNA Polymerase was from Stratagene and Phusion™, and High-Fidelity DNA Polymerase from New England Biolabs (Ipswich). All other reagents were of analytical grade and purchased from commercial sources.

### Cloning of genes for bacterial expression

The genes *lgt1* (lpg1368), *lgt2* (lpg2862), and *lgt3* (lpg1488) were amplified with PfuII Turbo from the genomic DNA of *L. pneumophila* strain Philadelphia-1 and cloned into a modified pET28a TEV as published [27]. The p4T2-403G (p5) plasmid, coding for GST-fused 45aa catalytic fragment of Tef1p was constructed as described previously [36]. The sequences of corresponding plasmids were confirmed by sequencing (GATC Inc., Konstanz, Germany).

### Cloning of genes for yeast expression

Initially, the coding sequence of yeast eEF1A with ∼500 nts upstream and downstream regions was amplified from *S. cerevisiae* chromosomal DNA using primers #518/#492 (Table S1), the product digested with BamHI/SalI restriction endonucleases and ligated into similarly digested pRS313 [52], producing plasmid p572. A C-terminal 6xHis-tag was inserted by amplification of the corresponding sequences from yeast chromosomal DNA with primers #649/#650 and #651/#492, digesting the products with NcoI/EcoRI and EcoRI/SalI and subsequently ligating them sequentially into pET28a, thus generating a plasmid p672. The final construct, encoding a C-terminally 6xHis-tagged yeast eEF1A was produced by replacing a part of p572 with the NcoI/SalI fragment from p672 thus generating plasmid p689. To clone the gene coding for the C-terminally 6-His-tagged G domain, the gene of yeast eEF1A with upstream and downstream regions (see cloning p572) was subcloned into YCpLac22 plasmid [53] using BamHI/SalI restriction sites (plasmid p553). Then, the SacI/XbaI fragment containing coding sequence of the G domain with upstream promoter region was ligated into the pBluescript KS (+) vector (p681). To attach a 6xHis-tag, the G domain-coding sequence was cut out from p681 using SacI/HindIII and ligated into pET28b (p682). Finally, the plasmid was amplified with the primers #16/#660, the amplicon digested with SacI/SalI and ligated into pRS423 vector [52] to generate the plasmid p710. To construct the C-terminally 6xHis-tagged yeast eEF1A S53A mutant, the region coding for the Ser-53-containing peptide was excised out of p553 with endonucleases SacI/ClaI and ligated into the pBC KS (+) vector, generating plasmid p574. QuikChange (Stratagene) site-directed mutagenesis technology was applied to this construct using primers #542/#543, generating the S53A substitution (plasmid p575). Subsequently, wild type coding region in the plasmid p689 (C-terminally 6xHis-tagged yeast eEF1A, see above) was substituted with the mutated sequence using SacI/ClaI insert of p575, to obtain the plasmid p726. For yeast transformation, standard genetic techniques were applied [54].

### Purification of recombinant proteins

*E. coli* BL21(DE3) transformed with the desired plasmid was grown in the LB broth supplemented with ampicillin or kanamycin on a shaker at 37°C until A_600_ = 0.8. Protein expression from the pET28-based plasmids was induced by 1 mM isopropyl-β-D-thiogalactopyranoside (Roth, Karlsruhe, Germany) for 4–5 h at 22°C, and for pGEX-based constructs with 0.2 mM isopropyl-β-D-thiogalactopyranoside at 37°C for 3 h.

Bacterial cells were harvested by centrifugation at 6,000 × g for 15 min, resuspended in lysis buffer (20 mM Tris–HCl (pH 7.4), 150 mM NaCl, 25 mM imidazole, 30 μg/ml DNase I, 10 mM β-mercaptoethanol, 1 mg/ml lysozyme and Proteinase Inhibitor Cocktail (Roche)) and lysed by French press or sonication. The cleared lysate was subjected to chromatography on a glutathione-Sepharose Fast Flow or nickel-equilibrated chelating Sepharose Fast Flow column according to the manufacturer's instructions (GE Healthcare). Bound proteins were eluted with 10 mM reduced glutathione, 0.5 M imidazole or thrombin treatment, depending on the construct used.

For the production of recombinant yeast 6xHis-containing protein, *S. cerevisiae* was grown overnight in a medium containing 0.67% yeast nitrogen base with ammonium sulfate without amino acids (Difco, USA), 100 µg/ml of L-leucine and 20 µg/ml of each L-tryptophan, uracil, and adenine, and 2% glucose. Yeast cells were disrupted by Oscillating Mill MM 400 (Retsch, Germany). Cleared extracts were subjected to HisTrap FF chromatography using Äkta Purifier (GE Healthcare). Yeast eEF1A (yEF1A) and mammalian eEF1A (mEF1A) were purified based on their interaction with His-tagged yEF1Bα as described previously [38]. In brief, yEF1Bα expressed in *E. coli* and purified on a HisTrap HP column was used to pulldown eEF1A from the commercial baker's yeast lysate or mouse liver tissue homogenate. The complex was applied onto a HisTrap HP column with eEF1A lysis buffer (100 mM Tris-HCl pH 7.6 at 6°C, 200 mM KCl, 5 mM MgCl2, 10% glycerol, 0.5 mM, β-mercaptoethanol, 20 mM imidazole, 0.1 mM PMSF, and protease inhibitor cocktail). The eEF1A·EF1Bα complex was eluted with a linear gradient of 20 to 250 mM imidazole. eEF1A was released from the complex by incubation with 100 µM GDP and applied onto Mono Q column (GE Healthcare, Freiburg, Germany) followed by further purification on a Resource S column (GE Healthcare) and dialysis against a final storage buffer (20 mM Tris-HCl (pH 7.6), 150 mM KCl, 5 mM MgCl_2_, 0.5 mM dithiothreitol, 15 μM GDP, and 25% glycerol). Glucosylated eEF1A was purified similarly.

### Aminoacyl-tRNA synthesis

Phe-tRNA^Phe^ was obtained by incubation of 5 µM purified yeast tRNA^Phe^ (Sigma, St. Louis, USA) and yeast phenylalanyl-tRNA synthetase (PheRS) (750 nM) [55] with 250 μM [^14^C]-phenylalanine (868 dpm/pmol) or 100 µM unlabeled phenylalanine in charging buffer (20 mM Hepes/KOH (pH 7.3), 2.5 mM spermidine trihydrochloride, 1 mM ATP, 5 mM MgCH_3_CO_2_, 1 mM DTT and 100 mM NH_4_Cl) for 20 min at 37°C. The extent of aminoacylation was determined by scintillation counting after trichloroacetic acid precipitation and filtration through GF/C filters. HPLC purified [^14^C]Phe-tRNA^Phe^ was prepared as described [56]. Histidyl-tRNA^His^ was prepared by charging yeast tRNA^His^ in the yeast tRNA mixture (Sigma) with histidyl-tRNA synthetase (HisRS) (MY Biosource, San Diego, USA). Ternary complex was formed after incubation of the components for 10 min at room temperature in the presence of 75 µM GTP, 0.1 mg/ml pyruvate kinase and 10 mM phosphoenolpyruvate. After ternary complex formation of yeast eEF1A, GTP and His-tRNA^His^, the remaining uncharged tRNA was digested with RNase A for 10 min at 4°C prior to glucosylation reaction.

### Glucosyltransferase assay

Yeast cell lysate used as crude substrate for Lgt was prepared by lysis of a pelleted yeast culture with an Oscillating Mill MM 400 (Retsch, Germany). Glucosylation was performed with 140 nM (if not otherwise noted) recombinant His-tagged Lgt1, 2, and 3 and crude eukaryotic cell extract or recombinant substrates (3 µM) in a total volume of 20 μl. The standard reaction proceeded at 30°C for 15 min in 20 mM Tris-HCl (pH 7.5), 150 mM NaCl, 1 mM MnCl_2_, and 10 μM UDP-[^14^C]glucose. The reaction was stopped by the addition of SDS-sample buffer and heating at 95°C for 5 min. Subsequently, samples were subjected to SDS-PAGE. Proteins were stained with Coomassie and radiolabeled bands were analyzed by PhosphorImaging and quantification with ImageQuant 5.2 (GE Healthcare, Freiburg, Germany).

### Aa-tRNA protection assay

Yeast eEF1A (20 µM) was glucosylated by 5 µM Lgt3 for 5 min at 37°C in the presence of 1 mM UDP-glucose, GTP, tRNA^Phe^, phenylalanine, PheRS, phosphoenolpyruvate, and pyruvate kinase as described above. Modified and unmodified yeast eEF1A was purified from TEV-cleaved Lgt3 by His-yEF1Bα affinity chromatography (see *Purification of Recombinant Proteins*). The formation of the ternary complex was obtained by incubation of 3 µM glucosylated or non-glucosylated yeast eEF1A with 1 µM [^14^C]Phe-tRNA^Phe^ from yeast, 1 mM GTP, 3 mM phosphoenolpyruvate, 1 mM ATP, 7 mM MgCl_2_ and 1% of pyruvate kinase in 70 µl of TAKM7 buffer (50 mM Tris-HCl (pH 7.5), 70 mM NH_4_Cl, 7 mM MgCl_2_ and 30 mM KCl) as described [33]. The reaction was carried out at 37°C and at various time points 2×5 µl of the reaction solution was precipitated by 10% trichloroacetic acid and transferred onto nitrocellulose filters. After washing with 5% trichloroacetic acid non-hydrolyzed [^14^C]Phe-tRNA^Phe^ was measured by scintillation counting.

### Cell culture

Raw 264.7 macrophages were cultivated in Dulbeccós MEM modified Eaglés medium supplemented with 10% fetal calf serum (FCS) and penicillin/streptavidine (Biochrom, Berlin, Germany). Cell lines were cultured at 37 °C in 5% CO_2_. The *Legionella* strains were grown on charcoal-yeast extract agar plates [31] or PP#3-liquid medium (per liter: 15 g proteose peptone no. 3 (Difco), 10 g ACES-buffer, 0.4 g L-cysteine H_2_O, 1 g α-ketoglutarate, (pH 6.9)). Kanamycin 25 µg/ml was applied where necessary.

### Isolation of in vivo glucosylated eEF1A

6x100 mm dishes RAW 264.7 macrophages (80% confluency) were incubated for 1 h in DMEM-medium without additives. *Legionella* strains were cultivated on charcoal plates and liquid medium to an OD_600_ of 2.0. Subsequently, RAW macrophages were infected with *L. pneumophila* strains indicated (multiplicity of infection = 100). After incubation for 5 h under cell culture conditions, macrophages were washed extensively and scraped off with lysis buffer (50 mM Tris (pH 7.4), 100 mM NaCl, 1 mM MgCl_2_, 1 mM PMSF and complete protease inhibitor mix (Roche)) and lysed by 10 strokes with a 1 ml syringe equipped with a G26 needle. After centrifugation (17.900 x g, 10 min), the post-nuclear supernatant was incubated with 100 µg His-tagged eEF1Bα for 15 min on ice followed by Ni^2+^-affinity chromatography. After elution with 500 mM imidazole, proteins were reduced by dithiothreitol treatment, alkylated with iodoacetamide, and separated by 12.5% SDS-PAGE. The corresponding bands of eEF1A were excised and analyzed by mass spectrometry.

### LC-MS/MS analysis

For in-gel digestion the excised gel bands were destained with 30% ACN, shrunk with 100% ACN, and dried in a Vacuum Concentrator (Concentrator 5301, Eppendorf, Hamburg, Germany). Digests with chymotrypsin was performed overnight at 25°C in 0.1 M NH_4_HCO_3_ (pH 8). About 0.1 µg of protease was used for one gel band. Peptides were extracted from the gel slices with 5% formic acid. All LC-MS/MS analyses were performed on a Q-TOF mass spectrometer (Agilent 6520, Agilent Technologies) coupled to a 1200 Agilent nanoflow system via a HPLC-Chip cube ESI interface. Peptides were separated on a HPLC-Chip with an analytical column of 75 µm i.d. and 150 mm length and a 40-nL trap column, both packed with Zorbax 300SB C-18 (5 µm particle size). Peptides were eluted with a linear acetonitrile gradient with 1%/min at a flow rate of 300 nl/min (starting with 3% acetonitrile). The Q-TOF was operated in the 2 GHz extended dynamic range mode. MS/MS analyses were performed using data-dependent acquisition mode. After a MS scan (2 spectra/s), a maximum of three peptides were selected for MS/MS (2 spectra/s). Singly charged precursor ions were excluded from selection. Internal calibration was applied. Mascot Distiller 2.3 was used for raw data processing and for generating peak lists, essentially with standard settings for the Agilent Q-TOF. Mascot Server 2.3 was used for database searching with the following parameters: peptide mass tolerance: 20 ppm, MS/MS mass tolerance: 0.05 Da, enzyme: “chymotrypsin” with 2 uncleaved sites allowed, variable modifications: Carbamidomethyl (C), Gln-> pyroGlu (N-term. Q), oxidation (M) and Hexose (ST). For protein identification SwissProt protein database was used.

## Supporting Information

Table S1 **Oligonucleotides, used for cloning of yeast eEF1A constructs.**
(DOC)
