# Supplementary material for: Aminoacyl-tRNA-Charged Eukaryotic Elongation Factor 1A Is the Bona Fide Substrate for Legionella pneumophila Effector Glucosyltransferases
Source: PLoS One. 2011 Dec 22;6(12):e29525. doi: 10.1371/journal.pone.0029525 (PMC3245282; doi:10.1371/journal.pone.0029525)
Supplement: Table S1 — Oligonucleotides, used for cloning of yeast eEF1A constructs. (DOC) [file pone.0029525.s001.doc]

**Supplementary** Table 1.

| ***ID number*** | ***Nucleotide sequence (5’-to-3’)*** | ***Plasmid*** |
| --- | --- | --- |
| #518 | CATATCACATAGGATCCAACAGGCG | p553, p572 |
| #492 | CATTACAGATAGCGTCGACCAAAGTATT | p553, p572, p672 |
| #649 | CCAACGCTCCATGGTACAAGG | p672 |
| #650 | TTATGAATTCAGTGATGATGATGATGATGTTTCTTAGCAGCCTTTTGAGC | p672 |
| #651 | GCTAAGAAAGAATTCGATTGATAAGAC | p672 |
| #16 | TAATACGACTCACTATAGG | p710 |
| #660 | CAGCAGTCGACTCAGCTTCCTTTC | p710 |
| #542 | TTAGGTAAGGGTGCTTTCAAGTACGCT | p575 |
| #543 | AGCGTACTTGAAAGCACCCTTACCTAA | p575 |
